# Phytoplankton Assemblage over a 14-Year Period in the Adriatic Sea: Patterns and Trends

**DOI:** 10.3390/biology13070493

**Published:** 2024-07-02

**Authors:** Sanda Skejić, Blanka Milić Roje, Frano Matić, Jasna Arapov, Janja Francé, Mia Bužančić, Ana Bakrač, Maja Straka, Živana Ninčević Gladan

**Affiliations:** 1 Institute of Oceanography and Fisheries, Šetalište Ivana Meštrovića 63, 21000 Split, Croatia; sanda@izor.hr (S.S.);; 2 Department of Marine Studies, University of Split, Ruđera Boškovića 37, 21000 Split, Croatia; frano.matic@unist.hr; 3 National Institute of Biology, Marine Biology Station Piran, 6330 Piran, Slovenia

**Keywords:** phytoplankton community, long-term data, diversity, chlorophyll *a*, neural gas analysis, solar radiation

## Abstract

**Simple Summary:**

This study of the phytoplankton community in the Adriatic Sea shows increasing species diversity over a 14-year period despite the effects of climate change in terms of increased sea surface temperature and increasing solar radiation in summer. Fluctuations in the dynamics of the different phytoplankton groups were observed in the studied area. The dominant groups were diatoms and phytoflagellates, but their proportions varied depending on the proximity to the coast as shown by the distribution of chlorophyll *a*.

**Abstract:**

Considering the role of phytoplankton in the functioning and health of marine systems, it is important to characterize its responses to a changing environment. The central Adriatic Sea, as a generally oligotrophic area, is a suitable environment to distinguish between regular fluctuations in phytoplankton and those caused by anthropogenic or climatic influences. This study provides a long-term perspective of phytoplankton assemblage in the central eastern Adriatic Sea, with 14 years of continuous time series data collected at two coastal and two offshore stations. The predominant phytoplankton groups were diatoms and phytoflagellates, but their proportion varied depending on the vicinity of the coast, as evidenced also by the distribution of chlorophyll *a*. In the coastal environment, the phytoplankton biomass was substantially higher, with a higher proportion of microphytoplankton, while small phytoplankton accounted for the majority of biomass in the offshore area. In addition, a decreasing trend in diatom abundance was observed in the coastal waters, while such trend was not so evident in the offshore area. Using a neural gas algorithm, five clusters were defined based on the contribution of the major groups. The observed increase in diversity, especially in dinoflagellates, which outnumber diatom taxa, could be a possible adaptation of dinoflagellates to the increased natural solar radiation in summer and the increased sea surface temperature.

## 1. Introduction

Studying the environment by detecting regularities and patterns helps to distinguish natural variability from potential signs of degradation and enables the prediction of future [1]. Predictions are more reliable as more observations are collected and integrated over time [2]. Collecting long-term data is an irreplaceable approach to unraveling causal mechanisms and distinguishing between anthropogenic and natural influences in complex marine ecosystems [3]. Here, phytoplankton long-term series represent one of the most valuable data collections in the Adriatic through in situ measurements, since phytoplankton plays a crucial role as an indicator of changes because of its rapid turnover and high sensitivity to environmental conditions. These characteristics make phytoplankton a key component of marine environmental monitoring programs, providing valuable insights into the health and functioning of aquatic ecosystems.

Long-term research is a fundamental tool not only for scientific purposes but also for addressing societal and policy needs. In the context of European policies, the Water Framework Directive (WFD, 2000/60/EC) designates phytoplankton as one of the “biological quality elements” used to assess the ecological status of coastal waters in Europe [4]. Additionally, the Marine Strategy Framework Directive (MSFD, 2008/56/EC) mandates Member States to evaluate the good environmental status of pelagic habitats [5]. This evaluation involves the analysis of a comprehensive set of indicators, related to plankton communities.

Obtaining phytoplankton datasets with detailed taxonomic information is difficult and time-consuming from various perspectives, and such time series are relatively rare compared to those of only chlorophyll *a*. Nevertheless, current long-term phytoplankton research and/or monitoring programs have deepened the knowledge about the behavior of phytoplankton in their reference areas through high-resolution taxonomic data, e.g., [6,7,8,9,10,11]. Such long-term studies also enable the assessment of the impact of regional- and basin-scale climate events on local ecosystem function [12]. In recent decades, significant meteoclimatic changes have overlapped with anthropogenic pressure in Adriatic and determined new trends in the trophic state of the marine environment and plankton communities [8,13,14].

Since many climate projections predict anomalous periods of increased air temperatures and decreased precipitation over the Adriatic in the future, especially in summer [15,16,17], it is important to characterize the phytoplankton dynamics in the central Adriatic in the last couple of decades. Therefore, the aim of this work was (1) to characterize the overall phytoplankton abundance from the existing high-resolution taxonomical dataset obtained at several long-term sampling station in the oligotrophic central Adriatic Sea, (2) to determine temporal and spatial patterns and variability in phytoplankton dynamics, (3) analyze the diversity of the studied stations to identify the most representative genera, and (4) to analyze the relation of species richness to increasing solar radiation and sea surface temperature.

## 2. Materials and Methods

### 2.1. Study Area and Datasets

**Figure 1:**
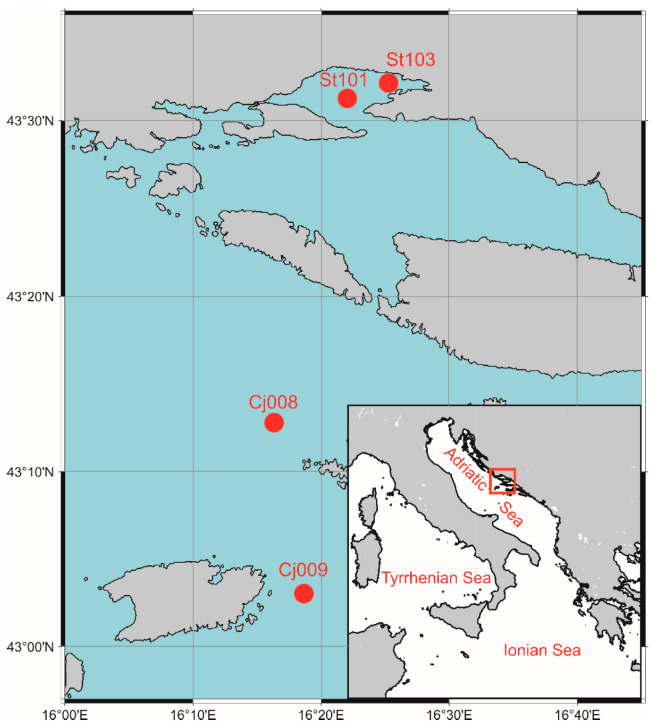
Map of the sampling coastal and offshore stations.

Phytoplankton samples were collected monthly at four stations in the central Adriatic Sea (Figure 1), providing a comprehensive 14-year-long dataset (2007–2020) of abundance and taxonomic composition. Sampling was carried with research vessels Bios (2007–2009) and Bios Dva (2010–2020), using the consistent methodology. Stations ST101 (depth 30 m) and ST103 (depth 17 m) are located in semi-enclosed Kaštela Bay, which was, prior to the installation of a wastewater collector in 2004, under considerable anthropogenic influence, but has since experienced a gradual transition towards oligotrophy [7,18]. The other two stations (CJ008 and CJ009) are located in the offshore central Adriatic along the transect towards the western Adriatic coast (Split–Monte Gargano), with depths of 75 m and 100 m, respectively (Figure 1).

### 2.2. Sampling and Analysis

Phytoplankton samples were collected as part of various national projects and the institutional long-term monitoring of the Institute of Oceanography and Fisheries. In total, 2289 phytoplankton samples were collected with Niskin bottles with monthly frequency (although with some gaps caused by funding discontinuity or technical problems, see Figure S1) along the vertical profile of each station at four to six depths in the water column (Table 1).

Samples for total chlorophyll *a* analyses were taken at all sampling stations at discrete depths (Table 1). Additional chlorophyll *a* samples were collected to determine the proportion of nanoplankton in the total phytoplankton biomass (size-fractionated chlorophyll *a*) at three–four depths of one coastal and one offshore station (Table 1).

### 2.3. Phytoplankton Analysis

Samples for phytoplankton community analysis (250 mL or 500 mL) were fixed with pre-filtered and neutralized formalin (with hexamethylene tetramine) to a final concentration of 0.4% in the sample. Identification and counting were carried out using inverted microscopes (LM) (Olympus IX50, IX51 and Leica BMI3000B) according to the Utermöhl method [19] using 25 mL sedimentation chambers for coastal waters samples and 100 mL for offshore samples. Counts were made at 400× magnification along 2 transects. Additionally, one half of the counting chamber was analyzed at 200× magnification to provide a more accurate estimate of the less abundant microphytoplankton taxa (>50 μm). Phytoplankton was identified to the lowest possible taxonomic level and then classified into major groups (diatoms, dinoflagellates, coccolithophores, phytoflagellates, and others). Abundances were expressed as cells per liter. Dinoflagellates were considered as an integral group including autotrophs, mixotrophs, and heterotrophs were included in the counts. All taxa were reviewed and checked against synonyms with reference to AlgaeBase: https://www.algaebase.org/ accessed on 21 June 2023). All organisms smaller than 10 μm with uncertain taxonomic affiliations (mostly around 3–4 μm) consisting of cryptophytae or other undetermined forms were assigned to the phytoflagellate group. Within the dataset, taxonomic determination is available to at least the genus level. The entire dataset consists of 34,079 entries of phytoplankton.

Over the study period, analysts performed the community analysis, using the same technique and regularly (yearly) participated in intercalibration tests (International Phytoplankton Intercomparison tests (IPI) since 2010. IPI operates according to the ISO standards 17043 [20]).

### 2.4. Chlorophyll a Analysis

Seawater samples for total chlorophyll *a* (500 mL) were filtered under low vacuum through glass GF/F filters (Whatman). Additional samples for size-fractionated chlorophyll *a* (500 mL) were pre-filtered through a 20 µm plankton net to exclude the microphytoplankton fraction and then filtered through GF/F filters. This filtration process made it possible to differentiate the proportion of micro- and nano-sized phytoplankton. Chlorophyll *a* concentration was determined fluorometrically from 90% acetone extracts following Ref. [21]. A Turner Trilogy laboratory fluorometer was used to measure the concentration.

### 2.5. Statistical Analysis

Data analyses were performed on three groups of phytoplankton parameters: (i) major groups abundance and distribution, (ii) species abundance and diversity, and (iii) chlorophyll *a*. At all three levels, the data were analyzed at long-term, monthly (seasonal), and vertical (depth) perspective, confronting coastal and offshore data.

Data analysis was performed in R (version 4.3.1) using RStudio (version 2023.06.1) [22]. Boxplots were drawn with following parameters: box representing interquartile range (25–75% percentile), thick line in the box representing the median, tails showing the range from minimum to maximum value without the outliers, and outliers are defined as any values >1.5 times the interquartile range over the 75th percentile or any values < 1.5 times the interquartile range under the 25th percentile. Indicator species analysis (ISA) was performed using the indicspecies package [23]. ISA was computed on surface sample data transformed to remove rare species (having less than 27 counts, or 5% of sample number) and with counts relativized by species maxima. The reported correlation between taxa and particular seasons corresponds to the indicator value (IndVal), computed with the multipatt function of the indicspecies package with default parameters.

Diversity indices were calculated in PRIMER 7 software [24]. We focused on the subset of species with unequivocally determined taxonomic identity, thus excluding the heterogenous groups (e.g., phytoflagellates). The following indices were calculated for each sample and then grouped for coastal and offshore stations for better visualization.

Principal coordinate analysis PCO was performed on surface data from all stations fourth root transformed using PRIMER 7 software. We considered all taxa, including phytoflagellates, data were averaged in a combined factor for area–season–year to reduce number of datapoints and the used distance among centroids (instead of averaging per individual factor). Seasons were defined as follows: winter Jan–Mar, spring Apr–Jun, summer July–Sept, autumn Oct–Dec, as previously determined for the Adriatic [25,26].

#### 2.5.1. Neural Gas Analysis

Neural gas (NG) analysis was used to reveal hidden structures in this complex phytoplankton dataset. The algorithm was implemented in the Python library NeuPy (new) with fixed parameters: step = 0.1; neighbor step = 0.001; maximum edge age = 50; number of iterations before adding a neuron = 100; aftersplit error decay rate = 0.5; error decay rate = 0.995; and minimum update distance = 0.2. The desired number of nodes was set to five—the number of resulting best matching units (BMUs). With the given temporal order, the dominant BMUs were determined using the smallest vector norm [27]. Using a neural gas algorithm, a large heterogeneous dataset of four hundred phytoplankton taxa was classified in terms of the contribution of four major groups (diatoms, dinoflagellates, coccolithophores, and phytoflagellates) to the overall community and distinguish five clusters (BMUs) (Table 2) according to the proportion of each group in the total phytoplankton abundance. BMUs are presented temporally (long-term and monthly) and spatially (along sampling depths).

#### 2.5.2. Solar Radiation and Sea Surface Temperature Analysis

Electromagnetic waves with ranges of 0.2–4 µm, covering the visible spectrum and responsible for photosynthesis, and near-infrared flux responsible for heating, that reach the sea surface layer, are represented with ERA5 reanalysis monthly mean incoming downward short-wave solar radiation. These data were downloaded from the Copernicus Climate Service data store [28].

Sea surface temperature (SST) was analyzed for latitude 43.0 N and longitude 16.5 E for monthly means, annual mean with linear trend. SST is the result of the Mediterranean Sea Physics Reanalysis oceanographic model and is downloaded from Copernicus Marine Service database (https://doi.org/10.25423/CMCC/MEDSEA_MULTIYEAR_PHY_006_004_E3R1, accessed on 11 June 2021). Trend of SST is 0.07 °C/year (0.7 °C/decade). The trend is calculated for period 2007–2020.

## 3. Results

### 3.1. Interannual and Seasonal Distribution of Main Phytoplankton Groups

**Figure 2:**
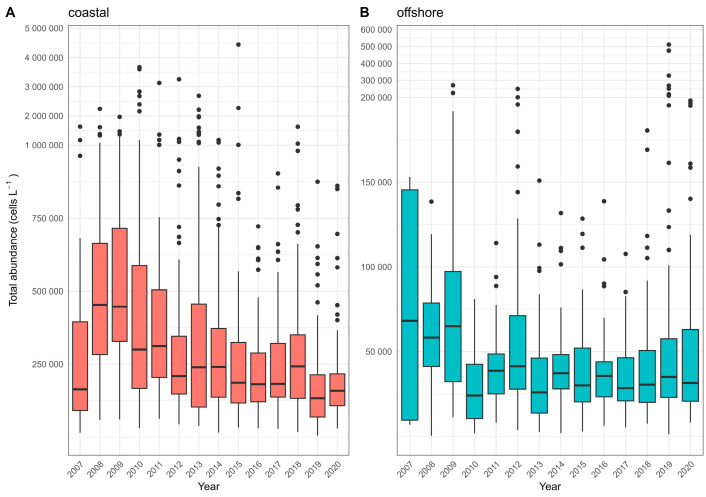
Long-term distribution (yearly biplots) of total phytoplankton abundance for (**A**) coastal and (**B**) offshore stations station.

The overall phytoplankton abundance showed a slight decline at the offshore stations and a more noticeable decline at the coastal stations from 2007 to 2020 (Figure 2). The total abundance in the coastal stations was around one order of magnitude higher (median 3.92 × 10^5^ cellsL^−1^, range 6.56 × 10^3^–4.44 × 10^6^ cellsL^−1^) as compared to that in the offshore stations (median 4.07 × 10^4^ cellsL^−1^, range 370–5.11 × 10^5^ cellsL^−1^).

**Figure 3:**
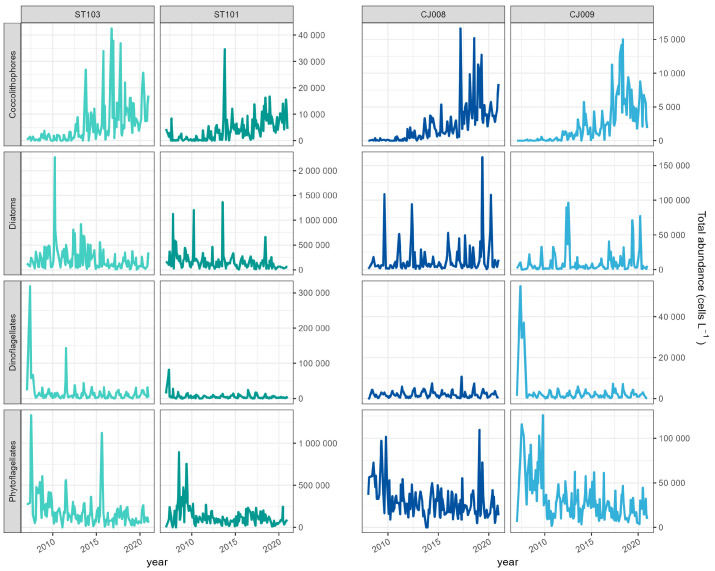
Abundance of major phytoplankton groups at coastal ST101, ST103 (**left panel**); and offshore stations CJ008, CJ009 (**right panel**) in the period 2007–2020.

The difference between phytoplankton abundance in coastal and offshore stations as well as its different trend is mainly attributable to diatoms and phytoflagellates (Figure 3). Diatoms displayed a decreasing trend at coastal stations, while there was no noticeable trend in abundance at offshore stations over the years (Figure 3). A trend of decreasing abundances was observed also for phytoflagellates regardless of the distance from the coast. In contrast to the diatoms, the coccolithophores exhibited increasing trend in abundance at all stations. The dinoflagellates had the lowest abundances among all groups, with occasional outbursts but no significant long-term trend at any of the stations (Figure 3).

**Figure 4:**
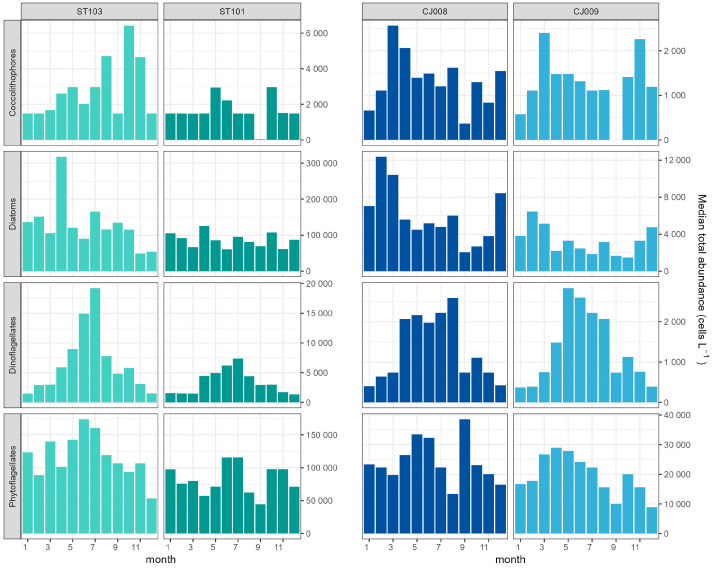
Monthly distribution of median abundances of major phytoplankton groups (including all depths) at coastal ST101, ST103; (**left panel**) and offshore CJ008, CJ009 (**right panel**) in the period 2007–2020.

Phytoplankton groups abundances showed dissimilarities between the coastal and offshore stations also in the seasonality (Figure 4). At coastal stations, diatoms did not exhibit a clear seasonality but rather isolated peaks, especially at ST103, for example a peak in spring (April) followed by a weaker summer peak in July. At offshore stations, the diatom seasonality was clearer, with higher abundances in late autumn/winter peaking in December and February.

The seasonal distribution of coccolithophores varied between stations, apparently unrelated to the vicinity of the coast (Figure 4). Station CJ009 showed an early peak in late winter/spring, while at CJ008, the highest values occurred in summer (July). Despite differences in timing, the abundances of coccolithophores in the coastal area show a notable increase in the late summer–autumn period (September–October).

Dinoflagellates were characterized by the most regular seasonal distribution, with abundance peaks observed at all stations in the warmer part of the year (May–October). As expected, miscellaneous phytoflagellates showed divergent seasonal patterns. At coastal stations, this group displayed a unimodal cycle, reaching peak values in August. In contrast, offshore stations exhibited an irregular seasonal pattern, suggesting that factors influencing phytoflagellate dynamics may differ between coastal and offshore environments (Figure 4).

### 3.2. Neural Gas Classification

Using a neural gas algorithm (NG), total phytoplankton dataset was reduced to five clusters (best matching units—BMUs) in which each cluster represents a particular proportion of the corresponding major group (diatoms, dinoflagellates, coccolithophores, and phytoflagellates, Table 2) for each sample of the dataset.

The first cluster (BMU1) was the most distinct, consisting of higher proportions of coccolithophores and dinoflagellates compared to the rest of the groups. BMU1 occurred only in 7% of the samples and could be considered an outstanding cluster due to its low occurrence. Another outstanding cluster was BMU5 due to a very high phytoflagellate ratio (83%) occurring the most frequently (29.4%). Clusters BMU3 and BMU4 were dominated by diatoms (54–81%), could be regarded as “standard” clusters (Table 2), and were more common at coastal stations.

**Figure 5:**
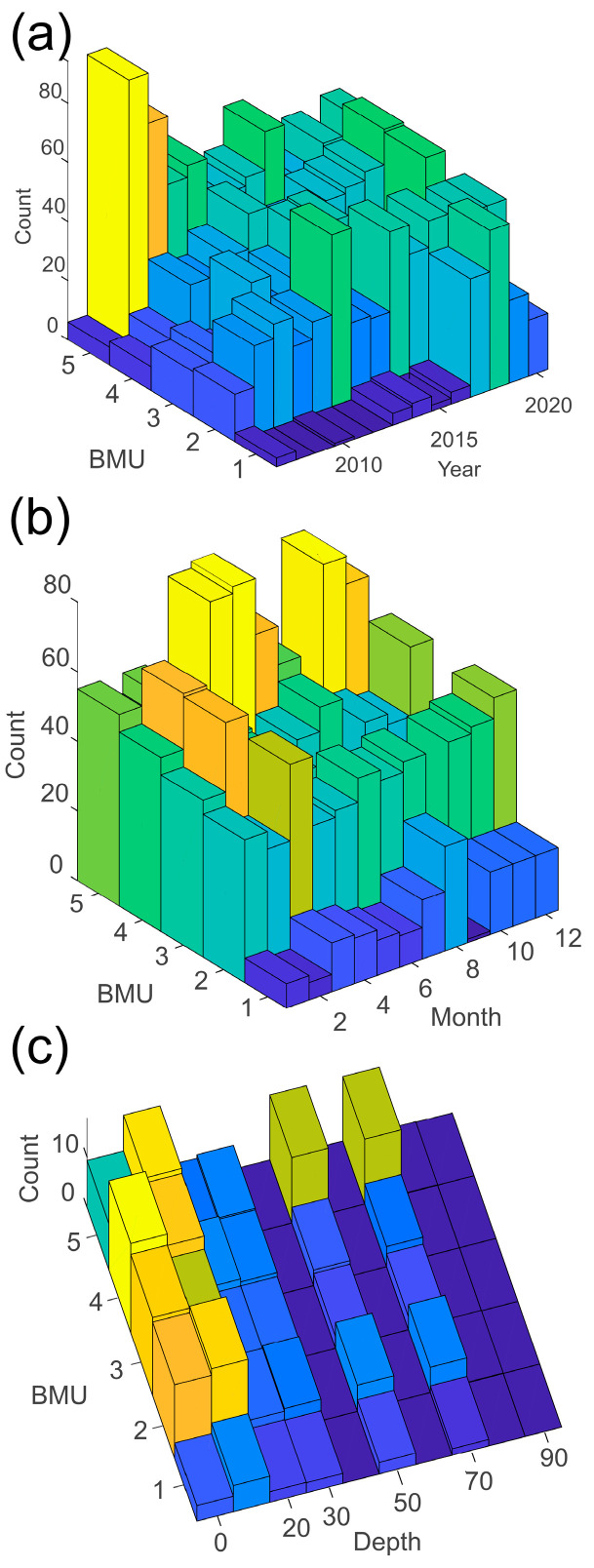
Distribution of best matching units (BMU) over (**a**) years, (**b**) months, and (**c**) depths.

There was no significant interannual alteration concerning the clusters of the total phytoplankton dataset (Figure 5a). The interannual trends in BMU distribution refer only to BMU5, which slightly decreased, and BMU1, which has increased in recent years, implying that the proportion of coccolithophores and dinoflagellates in the total phytoplankton community is rising.

The monthly distribution of BMUs (Figure 5b) showed that BMU5 was more frequent in the warmer season (May–October), while in colder months (particularly in February) the diatom-dominated clusters BMU3 and BMU4 prevailed.

The vertical distribution of BMUs (Figure 5c) showed that phytoflagellates dominate in deeper layers (offshore stations). On the contrary, when the diatoms increased, it was mainly in the surface layer, so BMU3 and BMU4 were mainly distributed in the surface layers. BMU5 describes the most common pattern in our samples (29.5%) with abundant phytoflagellates found mainly in offshore waters, while BMU3 and BMU4 with predominant diatoms are more commonly found at coastal stations.

With regard to the observed trends, diatom-dominated BMU3 (diatoms > 80%) showed a pronounced negative trend at coastal stations, implying that the proportion of diatoms in the coastal area is decreasing. Such a trend was not observed in the offshore area.

### 3.3. Phytoplankton Community Composition and Diversity Indices

Throughout the study period, 435 phytoplankton taxa were identified: 167 *Bacillariophyta*, 232 *Dinophyta*, 34 *Haptophyta*, 9 *Silicoflagellatae*, 1 *Crysophyceae*, 2 *Euglenophyta*, and 2 *Xantophyta*.

**Figure 6:**
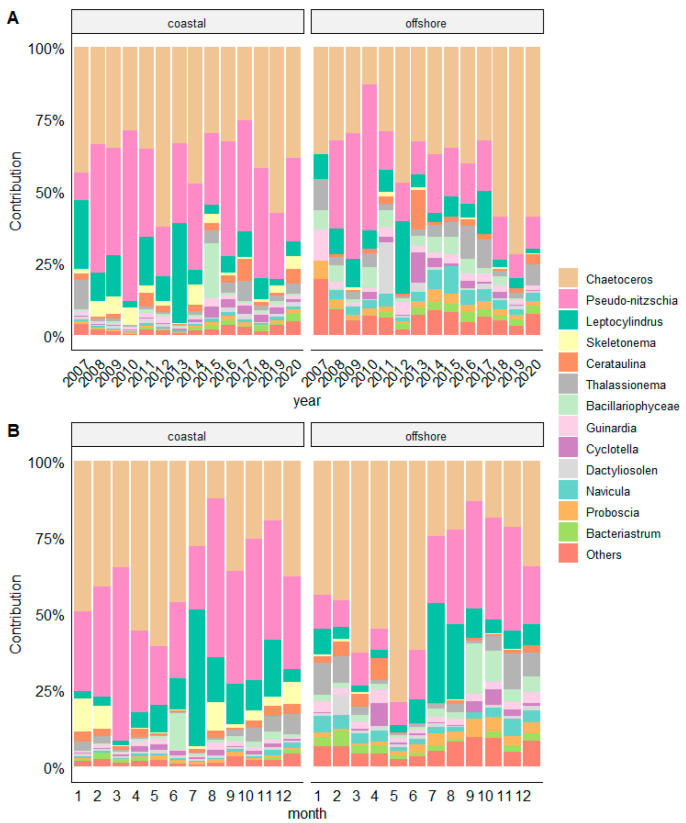
Relative contribution of diatom genera to all diatoms at coastal and offshore stations from a long-term (**A**) and seasonal perspective (**B**).

A slightly higher taxa richness was found in the coastal area (375 taxa) in relation to offshore stations (352 taxa). In total, 59 diatom and 48 dinoflagellate genera were recorded. The most abundant diatom genera were *Pseudo-nitzschia*, *Chaetoceros*, and *Leptocylindrus* (Figure 6). *Pseudo-nitzschia* showed a decreasing trend in abundance over the years (Figure 6A). As this genus is composed of many species with different seasonality, no regularity in temporal distribution could be detected (Figure 6B). The genus *Chaetoceros* was more or less evenly distributed throughout the year, with the highest abundances in spring. The genus *Leptocylindrus* showed consistently low abundance throughout the years, with the exception of a sharp increase in July 2013.

**Figure 7:**
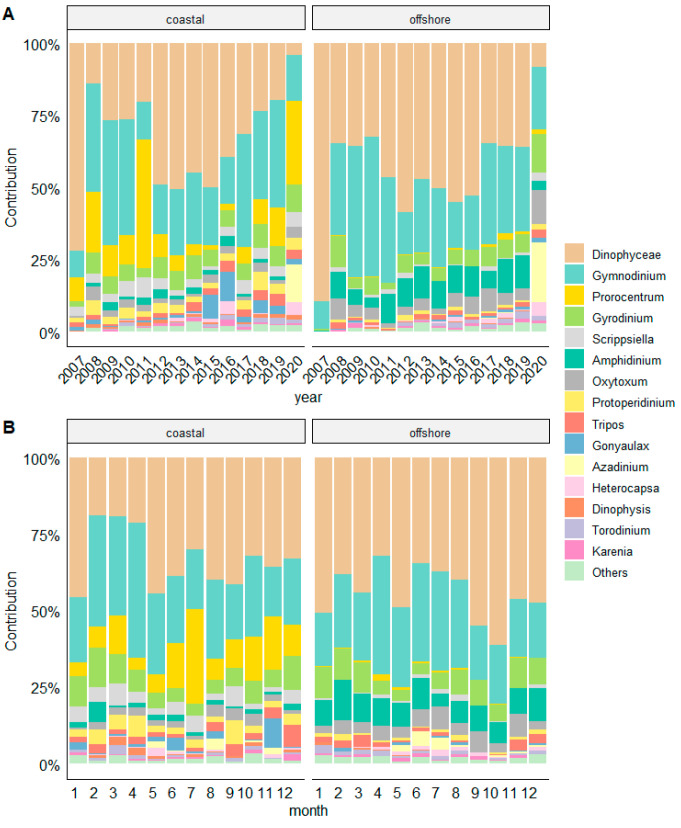
Relative contribution of dinoflagellate genera to all dinoflagellates at coastal and offshore stations from a long-term (**A**) and seasonal perspective (**B**).

As for dinoflagellates, their annual distribution displayed a uniform pattern, with genus *Gymnodinium* and unidentified dinoflagellates (<20 µm) exhibiting the highest abundances (Figure 7). Despite the overall low abundances, we recorded a high diversity within this group.

The extreme proliferation of phytoplankton (more than 10^6^ cells L^−1^) blooms was observed exclusively at the coastal stations. In particular, *Leptocylindrus danicus* thrived at both stations in autumn 2007 and summer 2013, reaching a maximum abundance of 2.05 × 10^6^ cells L^−1^ in July 2013. *Chaetoceros* dominated the spring bloom on several occasions (2010, 2012, 2013, and 2018), with a maximum abundance of 1.99 × 10^6^ cells L^−1^ in April 2010. However, *Chaetoceros* also occasionally reached high abundances in winter (March 2013, 1.58 × 10^6^ cells L^−1^) and summer (July 2012, 2.54 × 10^6^ cells L^−1^). *Pseudo*-*nitzschia* showed the highest abundances in winter (March 2010, 3.02 × 10^6^ cells L^−1^), and in summer (August 2013, 1.98 × 10^6^ cells L^−1^).

The relative contribution of genera prevailed at coastal stations were *Pseudo*-*nitzschia* (35.01% coastal vs. 15.35% offshore), *Leptocylindrus* (11.46% vs. 8.29%), *Skeletonema* (3.48% vs. 0.28%), *Prorocentrum* (14.22% vs. 0.72%), *Protoperidinium* (3.00% vs. 0.86%), and *Tripos* (2.07% vs. 1.24%). On the other hand, taxa more representative in offshore waters were *Proboscia alata* (1.93% vs. 0.65%), *Bacteriastrum delicatulum* (2.24% vs. 0.62%), *Navicula* spp. (2.98% vs. 0.61%), *Amphidinium* (8.03% vs. 1.65%), and *Oxytoxum* (4.75% offshore vs. 2.19% coastal) (Figure 7).

**Figure 8:**
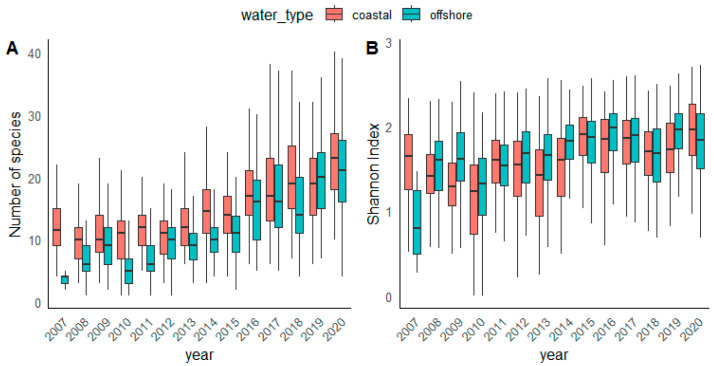
Interannual distribution of (**A**) species richness and (**B**) Shannon diversity at coastal and offshore stations.

The analysis of diversity revealed a consistent increasing trend in species richness at all stations, as depicted in Figure 8A. Notably, this trend was more pronounced in the offshore area. Both areas show significant increase in species richness confirmed by Mann–Kendall test (tau 0.408, *p* < 2.22 × 10^−16^ in Figure S2), as a similar pattern was observed for the Shannon index (Figure 8B).

Seasonal fluctuations in the diversity indices were less distinct (Figure S3). The highest species richness range in coastal waters was observed in autumn and in offshore waters in winter period. Both coastal and offshore stations displayed similar variations throughout the year, characterized by lower species richness during the summer months. The Shannon index showed the same pattern throughout the investigated area (coastal/offshore), although exhibiting a wider range of values at offshore stations. The winter–autumn period emerged as a time of increased Shannon diversity. Regarding the vertical distribution, species richness consistently decreased with depth, irrespective of station type (coastal or offshore) (Figure S4). In terms of Shannon diversity, this decrease was notably less pronounced in coastal area compared to offshore stations.

**Figure 9:**
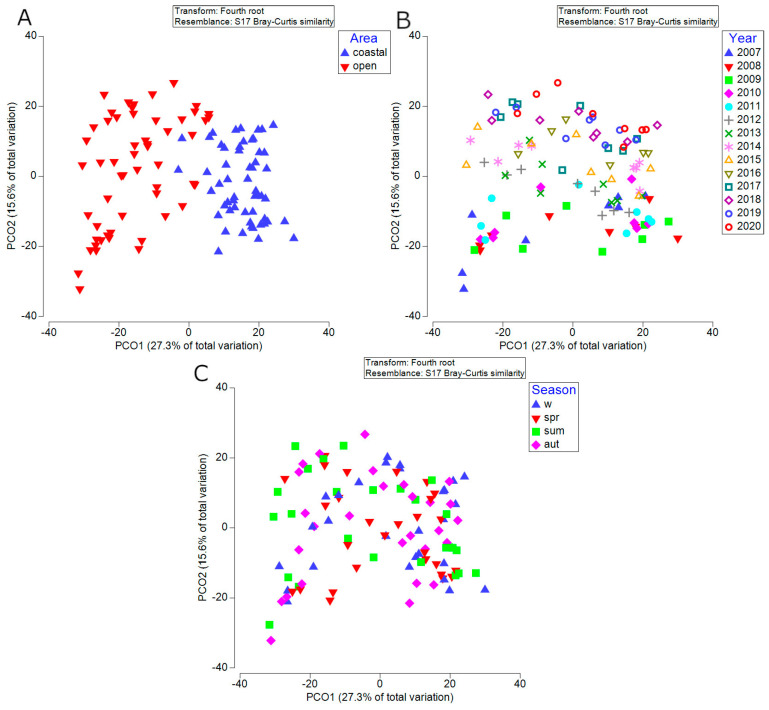
PCO biplots of samples based on the entire surface dataset of four investigated stations presenting different factors (**A**) area (**B**) year (**C**) season. Data were averaged using combined factor area–year–season.

The results of PCO corroborated the observed differences in phytoplankton community in coastal and offshore areas and long-term trends (Figure 9). A clear separation between the coastal and offshore area is visible along by the first PCO axis, which explained the highest proportion of variance (27.3%) (Figure 9A). The second PCO axis separated years (15.6% of variance). The year 2012 was of particular importance, as a boundary between two distinct settings: 2007–2012 and 2013–2020 (Figure 9B). As previously demonstrated, Figure 8A shows that since 2012, the increase in species richness is more noticeable. As for the seasons, no particular distinction was observed, suggesting that the seasonality was not distinguished (Figure 9C).

The indicator species analysis (ISA) computed for specific taxa across the entire dataset displays a correlation in form of their IndVal with particular seasons (Table 3). It is evident that certain species exhibited varying seasonality depending on their location. Spring season showed the highest consensus among taxa *Chaetoceros* spp., *Cyclotella* spp., *Dinophyceae, Tripos furca, Protoperidinium tuba, Gonyaulax polygramma, Gymnodinim simplex, Amphidinium* spp., *Gymnodinium* spp., and *Calyptrosphaera oblonga*. These taxa had high and statistically significant IndVal values in coastal areas, which mostly matched with their high IndVal values in open waters, although disparities exist. For example, *Dinophysis sacculus,* coccolithophores, and *Protoperidinium tuba* were not characteristic of spring in offshore waters, whereas they were in coastal. In contrast, *Rhizosolenia imbricata* and *Karenia* sp. were characteristic in open waters (IndVal 0.423 and 0.384, respectively), but not in coastal. The coccolithophore *Rhabdosphaera clavigera* had the highest IndVal (0.478) during the summer in offshore waters and was also a characteristic summer species in coastal waters with IndVal = 0.397. Other coccolithophores such as *Syracosphaera pulchra* were characteristic of the winter in coastal waters (IndVal 0.513), but not in offshore waters, while *Calciosolenia brasiliensis* was characteristic of winter in offshore waters (0.501). Of the 10 taxa characterizing autumn in coastal waters, only two of them, *Thalassionema nitzschioides* and *Chaetoceros peruvianus*, were also characteristic of autumn in offshore waters (Figure 6).

**Figure 10:**
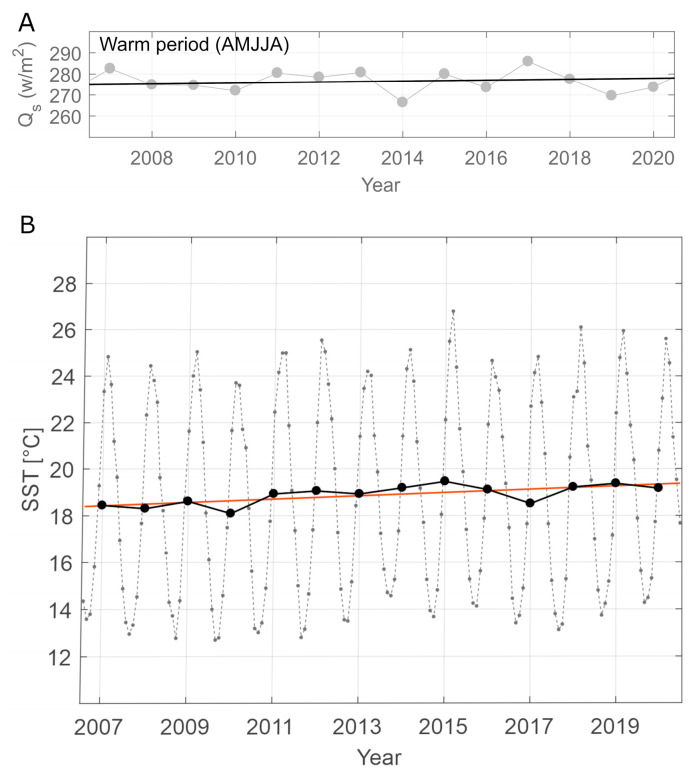
(**A**) Mean values of incoming short-wave radiation (Qs) from ERA5 analysis for the latitude 43.0 N and longitude 16.5 E for warmer part of the year (April–August) during 2007–2020. (**B**) Sea surface temperature (SST) at latitude 43.0 N and longitude 16.5 E for monthly means (gray dotted line), annual mean (black dots and line) with linear trend (orange line) during 2007–2020.

The most diverse phytoplankton group—dinoflagellates, with 232 taxa showed the most pronounced seasonal pattern with increasing abundance during the warmer period from April until August. This coincided with increased insolation trend calculated for the same months during the entire study period (Figure 10A). Moreover, an increasing trend in sea surface temperature (SST) of 0.07 °C per year was calculated over the sampling period (Figure 10B). Statistically significant correlation was calculated for dinoflagellate species richness and insolation (Mann–Kendall test, tau = 0.223, *p* < 2.2 × 10^−16^, Figure S5) and SST (Mann–Kendall test, tau = 0.175, *p* < 2.22 × 10^−16^, Figure S6).

### 3.4. Chlorophyll a

**Figure 11:**
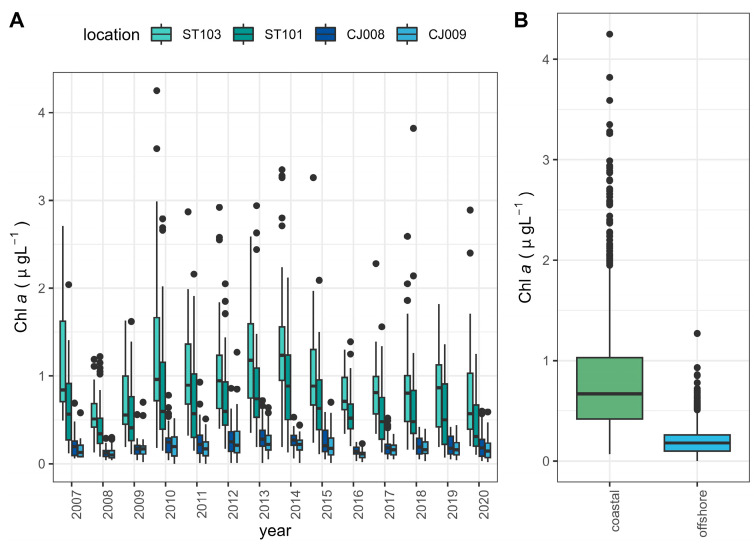
(**A**) Distribution of total chlorophyll *a* trough investigated period for four investigated stations. (**B**) Comparison of chlorophyll *a* at coastal and offshore stations.

Chlorophyll *a* concentration during the study period ranged from 0.00 to 4.25 µgL^−1^ in the dataset. The coastal stations showed significantly higher values, ranging from 0.07 to 4.25 µgL^−1^, than the offshore stations with a range of 0.00–1.27 µgL^−1^ (Wilcoxon rank sum test with continuity correction, *p*-value ≤ 2.2 × 10^−16^) (Figure 11A,B). We did not observe any particular trend at either coastal or offshore stations (Figure 11A).

**Figure 12:**
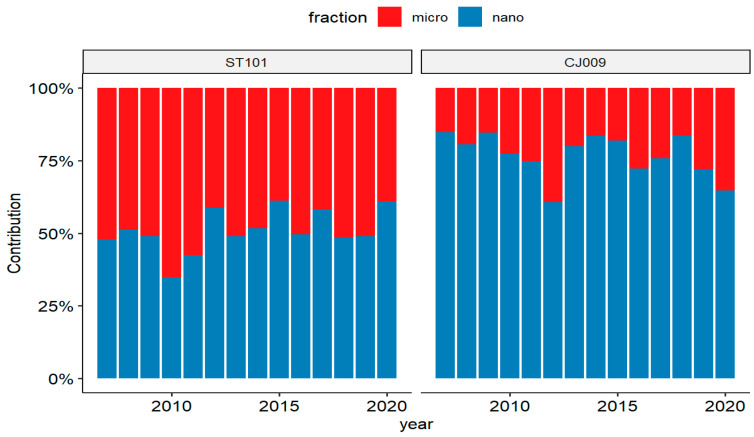
Relative proportions of phytoplankton size-fractionated Chl *a* at coastal (ST101) and offshore (CJ009) stations.

With regard to the relative proportion of phytoplankton size fractions (microphytoplankton >20 µm and nanoplankton <20 µm) for representative stations, it was evident that the nanophytoplankton prevailed at the offshore station CJ009 throughout the study period (Figure 12), whereas it was surpassed by the microphytoplankton at coastal station ST101.

**Figure 13:**
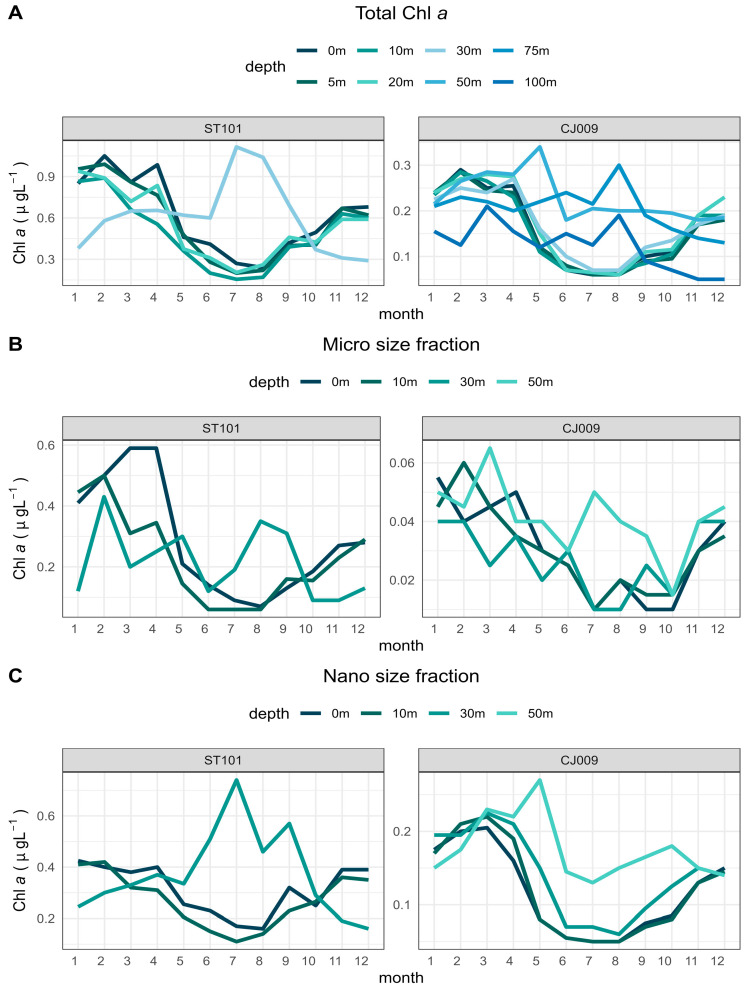
Chlorophyll *a* distribution by depth (**A**) total (**B**) microphytoplankton and (**C**) nanophytoplankton.

At station ST101, chlorophyll *a* values displayed a more or less typical yearly cycle for both size fractions with maximum values occurring in late winter/early spring and in late autumn period (Figure 13). An exception was observed at the depth of 30 m, where the Chl *a* exhibited almost an opposite cycle with an increase in summer. A similar dynamic was observed at the offshore station CJ009, but with more consecutive peaks in the micro-fraction and without a summer peak in nano-plankton. 

The seasonal differences of phytoplankton biomass (Chl *a*) were clearly distinguished and confirmed by Kruskal–Wallis test, as shown in (Figure S6), which showed that only spring and autumn exhibit similarity.

## 4. Discussion

### 4.1. Phytoplankton Groups—Interannual and Seasonal Distribution

This study provides a rare long-term perspective of phytoplankton diversity and abundance in the central eastern Adriatic Sea, with 14 years of continuous time series data collected in trophically distinct environments. As previous investigations from the same area mainly focused on primary production and biomass [14,29] or short-term studies [30], this research deepens into the phytoplankton seasonal and interannual patterns at high taxonomic resolution.

The studied environments in the central eastern Adriatic represent a gradient of depth, salinity, and nutrient content, with coastal stations generally showing moderate anthropogenic influence [31] and the offshore stations representing unimpacted sites [29]. These differences were expected to support different phytoplankton communities, particularly in terms of abundance and vertical variability.

The results showed that, overall, the dominant phytoplankton groups were phytoflagellates, which dominated in abundance throughout the study period, except during diatom blooms in coastal waters. Similar dominance of small flagellated forms of phytoplankton was also documented for the northern Adriatic for various time spans and locations in the last 30 years [25,26,32,33,34,35].

Unlike the trend reported by Rousseaux and Gregg [36], which indicated a significant decrease in diatom abundance on a global scale and Derolez et al. [37], who stated similar for the western Mediterranean area, our data confirmed such a tendency for coastal waters, while it was not so emphasized in offshore area. This inconsistency underlines the importance of considering regional small-scale variability in controlling factors such as changes in stratification period and photosynthetically active solar radiation when interpreting the phytoplankton patterns. Long-term studies by Marić et al. [6], Mozetič et al. [33], and Totti et al. [8] also emphasized the local Adriatic influences and the time span of evaluation for the resulting diatom abundance trends.

In contrast to diatoms, coccolithophores exhibited a distinct upward trend, particularly offshore. The occurrences of coccolithophores in the Adriatic Sea have been historically associated with periodic intrusions of more saline water from the eastern Mediterranean. This contradicts the recent findings by Ljubimir et al. [38], who suggested that higher coccolithophore proportions were observed during the anticyclonic phase of the BiOS mechanism [39] and lower salinity in the Adriatic.

The seasonal distribution of coccolithophores varied within this study, with offshore stations displaying higher abundances in late winter/early spring, while coastal stations exhibited peak abundances in autumn. Totti et al. [8] observed a shift towards lower winter abundances and a summer peak of coccolithophores in the recent period at a coastal station at the southern border of the northern Adriatic. In the same area, but at an offshore station, Neri et al. [10] reported similar coccolithophore dynamics to the one observed at offshore stations in our study. This regionally very diverse coccolithophore dynamics probably reflect their ability to proliferate in various environments, including coastal areas and estuarine systems and adds complexity to their ecological dynamics [40,41,42]. However, despite their efficiency in utilizing low nutrient concentrations, coccolithophores do not emerge as major contributors to the phytoplankton community ([36], this study). Nevertheless, the observed increase in coccolithophores in the Adriatic resonates with the global findings of Rousseaux and Gregg [36], who reported a significant rise in coccolithophore abundances in the North Atlantic and North Pacific.

In our study, only the dinoflagellates showed a pronounced seasonal cycle, but their low abundances are not comparable with the values from the 1970s–1990s, when the dinoflagellates outnumbered or equaled the diatoms [14], causing blooms. Changes in the DIA/DINO ratio have been used as an indicator of environmental change [29,43], but seem to have become a less suitable indicator for the Adriatic Sea in recent decades due to general prevalence of diatoms after 2000. Diatoms prevailed in abundance in all seasons, even during summer. This change in DIA/DINO ration could be due to oligotrophication in recent decades in the Adriatic [44].

Diatoms and dinoflagellates contribute the most to the microplankton size fraction, which showed a dominance in the nearshore area, while the smaller phytoplankton (<20 μm), mainly composed of phytoflagellates, coccolithophores, and picophytoplankton prevailed in offshore stations, as previously reported by Ninčević et al. [45]. Field studies have consistently shown that coastal nutrient-rich conditions favor the dominance of large sized phytoplankton, while oligotrophic conditions of the Adriatic open waters favor the prevalence of smaller phytoplankton. This is also in line with observations derived by satellite data, which point to the more or less equal contribution of nano- and picophytoplankton in the area of interest, together constituting around 80% of phytoplankton biomass [46].

In terms of seasonal distribution, the pattern of higher values in autumn/winter with the peak in March and lower values in late spring/summer in the upper water layer conform well with the “no bloom” cluster of D’Ortenzio and Ribera D’Alcalà [47] typical for the eastern Adriatic Sea. The different seasonal behavior of chlorophyll *a* in the deeper water layers, observed in both analyzed size fractions can be attributed to the development of deep chlorophyll maximum (DCM). According to Ninčević et al. [45], the DCM in the central Adriatic reaches the peak during spring with diatom dominance. Photoacclimation as a response of phytoplankton to lower light intensities in the deeper layers could also contribute to higher chlorophyll *a* concentration [48], which in this case does not necessarily reflect a biomass peak.

The decrease in total abundance in both near- and offshore areas was not paralleled in the chlorophyll *a* trend. Chlorophyll *a*, rather, displayed interannual fluctuations within the range previously documented for this area [14] and with significant differences between coastal and offshore areas reflecting the trophic gradient from the coast towards the open sea [49]. However, a maximum in yearly chlorophyll *a* was observed in the period 2012–2014 that could be connected to a deep mixing event in the Adriatic Sea in winter 2012, caused by extreme weather conditions that triggered record-breaking densities and nutrient enrichment in the upper water layers [50]. Indeed, the diatoms, which could have the major contribution to chlorophyll *a* during blooms, peaked in 2012 in the offshore area. The central eastern Adriatic ecosystem could also experience long-term trends in chlorophyll *a* concentration, as documented by several studies [49,51], that went undetected by the present study. Detecting signals of climate change that go beyond natural variability requires even longer time series of chlorophyll *a* [14,52], which have recently been supplemented by ocean color data from satellites [53].

Summarizing, the long-term decline in phytoplankton abundance, more pronounced in coastal areas, was primarily due to a decrease in diatoms and phytoflagellates as they are the most abundant groups, while coccolithophores were increasing. Dinoflagellates increased in number of taxa. These trends showed spatial and seasonal variability. Seasonally, diatoms exhibited a distribution typical for temperate areas, dominating or co-dominating with phytoflagellates in the colder months characterized by a mixed water column, as shown by the peaks in BMU3 and BMU4. In the warmer months, on the other hand, there was an increase in coccolithophores and dinoflagellates, which corresponds with the increased occurrence of BMU1. In spatial terms, diatoms were more strongly represented at the surface, while phytoflagellates dominated in the deeper layers. The upward trend in coccolithophores and dinoflagellates extended from the surface to the thermocline, suggesting a significant shift in the composition of the phytoplankton community in the different water layers.

### 4.2. Phytoplankton Community Composition and Diversity

The interannual dynamics of the phytoplankton community revealed taxonomic variations, with genera *Pseudo-nitzschia* and *Chaetoceros* contributing the most to the diatom community throughout the study period. Both genera are important phytoplankton builders exhibiting different seasonality: while *Chaetoceros* was typical for spring period, *Pseudo-nitzschia* thrived mainly in winter, consistent with previous findings [9,54]. The genus *Pseudo-nitzschia* comprises about 60 species that have different preferences in terms of environmental conditions. For example, Bernardi Aubry et al. [55] found that *Pseudo-nitzschia* dominated throughout the year and peaked in August, suggesting the opportunistic nature of this genus. Blooms of *Pseudo-nitzschia* species in different parts of the Adriatic Sea were observed in different seasons: winter/autumn [56,57], summer/autumn [6,58]. Long-term phytoplankton observations suggest the sustained prevalence of *Pseudo-nitzschia* is possibly linked to rising salinity and temperature [59]. Indeed, the ecological niche of *Pseudo-nitzschia* is characterized by high irradiance, high water temperatures, and salinity alongside low nutrient concentrations and turbidity [60]. On the other hand, *Pseudo-nitzschia* exhibited remarkable adaptability to changing light and temperature conditions even in reduced sunlight in winter [61].

The consistent occurrence of *Chaetoceros* spp. throughout this study supports its characterization as a cosmopolitan species. The studies by Mozetič et al. [44] and Marić et al. [6] provided additional evidence for the increased occurrence of various *Chaetoceros* species in recent decades, especially during the late winter and early spring. *Chaetoceros* is frequently reported from coastal regions along the eastern Adriatic coast, as documented by Bužančić et al. [30]. Another abundant diatom genus in our study was *Skeletonema*, which was more typical for coastal waters. *Skeletonema* is a globally important phytoplankton constituent [62] that has caused regular winter blooms in the northern Adriatic [6,8]. However, the recent decline in abundance of this genus, as observed in this study, and also in the northern Adriatic [8,34], has been linked to the decrease in nutrients [32].

Compared to diatoms, the dinoflagellate community composition was more variable between years, but followed typical seasonal pattern of increasing abundances from May to August. For example, *Gymnodinium* spp. together with small unidentified dinoflagellates were the most numerous representatives of the dinoflagellate community in coastal and offshore stations, particularly numerous in July. In the coastal zone, the abundance *Prorocentrum* also increased during summer. *Prorocentrum* was occasionally found in large numbers, mostly attributed to *P. cordatum.* The importance of dinoflagellates in spring and summer during our study was also corroborated by ISA, since different dinoflagellate taxa resulted the most important constituents of the phytoplankton community during these seasons with significant IndVal values, regardless of the distance from the coast. The importance of dinoflagellates should be therefore searched in their high diversity, not in the significant contribution to abundance and biomass. Indeed, according to Bernardi Aubry et al. [63], the importance of dinoflagellates in the communities was generally low (1% of abundance, 13% of biomass), with significant presence only in summer. However, Totti et al. [8] observed a shift, revealing a more relevant contribution of dinoflagellates in the spring community (e.g., *P. cordatum* and *Noctiluca scintillans*) in the recent years, which is in line with our findings.

Potentially toxic dinoflagellate genera, such as *Alexandrium, Dinophysis*, and *Karenia* species, were not very abundant during our study, but occurred regularly in small numbers. Although *Lingulodinium polyedra* in previous periods formed blooms [29,64], during our study, it was not the case.

Although the two studied areas of the central Adriatic exhibited some similarity, the coastal vs. offshore environment determined important differences in the seasonality of the species. While in the coastal areas different seasons had a similar number of characteristic taxa, although different in structure (dinoflagellates taxa characterizing spring and summer, diatoms autumn and winter), the offshore stations had many more characteristic taxa for spring and winter, and just a few for summer and autumn. For example, 10 taxa characterized the summer in the coastal region, but of these, only the coccolithophore *Rhabdosphaera clavigera* was identified as characteristic for summer at the offshore station. Moreover, many of the taxa characteristic for the autumn in the coastal zone occurred later in winter in offshore environment. Therefore, given the differences in hydromorphological characteristics. On the contrary, the genus *Pseudo-nitzschia*, which occurred in high abundances in all seasons, was not characteristic of any season.

In our data, a certain change in the number of phytoplankton taxa as well as in the structure of the community is recognizable. There are studies that indicate significant changes over time due to climate change. For example, Ajani et al. [65] conducted a study covering nine decades revealing significant shifts in phytoplankton community composition associated with the ocean warming trend of 1.8 °C.

The time series analysis revealed a remarkable diversity trend in the central Adriatic, indicating an increase in the richness of phytoplankton genera (species) at all stations with a simultaneous decrease in phytoplankton abundance. As in all time series, the increasing diversity could be influenced by improved taxonomic knowledge of analysts, but our results were confirmed by consistent findings in Slovenian [35] and Italian waters [10], suggesting that this pattern extends beyond national boundaries. In addition, studies in the western Mediterranean [37] and in Helgoland area [66] support the recent observation of the shift towards higher taxonomic diversity of phytoplankton, the latter attributing the increasing diversity to increased system stability. This could be aligned with changes indicating a transition towards more oligotrophic conditions and reduced pollution impact, attributed to improved wastewater management practices observed for the northern Adriatic [33,44]. Similar observations were made by Marasović et al. [29], who investigated phytoplankton groups and primary production in the central Adriatic in the 2000s compared to the 1980s–1990s. Authors found that changes at the coastal stations cannot be solely attributed to human influences. While previous interpretations focused on anthropogenic pollution, Marasović et al. [29] discovered that similar changes occurred in open waters, suggesting broader, possibly global shifts, potentially linked to climate change. This trend aligns with other Adriatic studies [8,10,14,26,35].

An increasing trend in phytoplankton diversity was also observed by Sarker et al. [66] for the Helgoland dataset over several decades, who linked increased diversity to low ecosystem variability. The authors claimed that the 1980s was a period of high system variability, and although the temperature has been increasing and some extreme events occur, the Helgoland ecosystem is generally less exposed to extreme climate variables than in the 1980s [66].

While the prevailing opinion of many authors suggests that oligotrophication and rising temperature associated with climate change could lead to a reduction in the number of phytoplankton species and favor smaller species [33,67,68,69,70], Fu [71] challenges this consensus by suggesting that phytoplankton communities may evolve towards larger-sized populations in the context of climate change-induced warming of seawater. Fu’s argument considers that certain larger phytoplankton species have adaptive advantages under the predicted conditions of climate change, for example, a competitive advantage in utilizing resources and light capturing. Additionally, larger phytoplankton may be more resilient to changes in seawater chemistry, such as acidification, compared to smaller and more delicate species. In addition, Rousseaux and Gregg [36] found that in the northern mid-latitudes (north central Pacific and Atlantic), the decline in nutrients lead to a decline in smaller phytoplankton (i.e., cyanobacteria and coccolithophores) suggesting that nutrient concentrations could be so low that even the cyanobacteria, which are characterized by very low nutrient requirements, were negatively affected.

In our study, the observed increase in phytoplankton diversity can be partly attributes to an increase in dinoflagellate taxa, which outnumber that of diatoms. High dinoflagellate diversity was reported also for long-term studies in different regions of the Adriatic Sea [7,8,10,35,38,72]. This increased diversity of dinoflagellates together with the pronounced seasonal pattern may be a potential adaptation to the increased solar radiation during summer months and increased SST. The correlation between increased solar radiation in warmer period and alterations in phytoplankton communities, as shown in our study, underlines the complicated relationship between environmental factors and phytoplankton dynamics. The influence of light absorption on phytoplankton growth, as demonstrated by Alvarez et al. [73], emphasizes the importance of considering various factors that influence light availability in natural environments [74]. Factors such as wind, turbulence, upwelling, and waves play an important role in modulating light fluctuations. Regarding temperature, the documented increase in SST in the central Adriatic is consistent with previous studies [75] and likely contributes to the observed trends in species richness. The SST increase has even accelerated from 2008 onwards, and showed a linear trend of 0.013 °C, affecting the microbial food web [76]. These results are in accordance with the general finding that species richness increases with increasing temperatures up to a certain threshold before decreasing [77,78,79,80].

To outline, the observed rise in phytoplankton diversity can be tentatively attributed to climate change-related processes. Still, although both near- and offshore areas experienced a similar trend, important differences between both phytoplankton communities remained, driven by different prevailing natural conditions.

## Figures and Tables

**Table 1:** Sampling stations and depths for the analysis of phytoplankton community and chlorophyll *a* during the investigated period.

	Station	Sampling Depth (m)	Parameter
Coastal	ST103	0, 5, 10, 20 0, 5, 10, 20	Community composition Total chl *a*
	ST101	0, 5, 10, 20, 30 0, 5, 10, 20, 30 0, 10, 30	Community composition Total chl *a* Size fractionated chl *a*
Offshore	CJ008	0, 10, 30, 50, 75 0, 10, 30, 50, 75	Community composition Total chl *a*
	CJ009	0, 10, 30, 50, 75, 100 0, 10, 30, 50, 75, 100 0, 10, 30, 50	Community composition Total chl *a* Size fractionated chl *a*

**Table 2:** Characteristics of clusters (best matching units—BMU) identified by NG analysis: percentage contribution of major phytoplankton groups and frequency of occurrence in the dataset. (Abbreviations: DIAT—diatoms; COCCO—coccolithophores; DINO—dinoflagellates; PHYTO—phytoflagellates; FREQ—frequency).

CLUSTER	DIAT	COCCO	DINO	PHYTO	FREQ (%)
BMU1	15.25	29.60	15.06	40.09	7
BMU2	29.37	4.22	4.09	62.32	22.7
BMU3	81.87	2.92	3.02	12.19	18.9
BMU4	54.84	4.41	3.16	37.60	22.6
BMU5	8.69	2.88	3.63	84.80	29.4

**Table 3:** List of phytoplankton taxa characterized by the highest IndVal for each season in the 2007–2020 period. Ind Val values for coastal and offshore stations indicated * are significant at *p* < 0.05; those in ** are significant at *p* < 0.01. (GROUP abbreviations: DIAT—diatoms; COCCO—coccolithophores; DINO—dinoflagellates; PHYTO—phytoflagellates; SILICO—silicoflagellates).

Phytoplankton Taxa	Group	Coastal			Offshore		
		Season	IndVal	Significance	Season	IndVal	Significance
*Chaetoceros* spp.	DIAT	**spring**	0.583	*	**spring**	0.601	**
*Calyptrosphaera oblonga*	COCCO	**spring**	0.478	**	**spring**	0.554	**
*Gonyaulax polygramma*	DINO	**spring**	0.421	**	**spring**	0.290	*
*Gymnodinium simplex*	DINO	**spring**	0.348	**	**spring**	0.295	*
*Amphidinium* spp.	DINO	**spring**	0.329	**	**spring**	0.460	**
*Gymnodinium* spp.	DINO	**spring**	0.581	**	**spring**	0.596	**
*Dinophysis sacculus*	DINO	**spring**	0.377	**			
*Coccolithophyceae*	COCCO	**spring**	0.474	*			
*Cyclotella* spp.	DIAT	**spring**	0.450	*	**spring**	0.426	**
*Tripos furca*	DINO	**spring**	0.343	*	**spring**	0.441	**
*Protoperidinium tuba*	DINO	**spring**	0.312	*			
*Dinophyceae*	DINO	**spring**	0.472	*	**spring**	0.540	*
*Gyrodinium fusiforme*	DINO	**spring**	0.432	*	**summer**	0.437	*
*Scrippsiella trochoidea*	DINO				**summer**	0.375	**
*Proboscia alata*	DIAT	**summer**	0.639	**	**spring**	0.488	*
*Prorocentrum triestinum*	DINO	**summer**	0.510	**			
Phytoflagellates		**summer**	0.648	**			
*Leptocylindrus danicus*	DIAT	**summer**	0.663	**			
*Guinardia striata*	DIAT	**summer**	0.514	**			
*Rhabdosphaera clavigera*	COCCO	**summer**	0.397	*	**summer**	0.478	**
*Dactyliosolen fragilissimus*	DIAT	**summer**	0.420	*			
*Oxytoxum laticeps*	DINO	**summer**	0.236	*	**spring**	0.308	*
*Protoperidinium steinii*	DINO	**summer**	0.298	*	**spring**	0.281	**
*Karenia* sp.	DINO				**spring**	0.384	**
*Thalassionema nitzschioides*	DIAT	**autumn**	0.604	**	**autumn**	0.392	*
*Chaetoceros peruvianus*	DIAT	**autumn**	0.410	**	**autumn**	0.245	*
*Chaetoceros diversus*	DIAT				**autumn**	0.340	*
*Dictyocha fibula*	SILIC	**autumn**	0.434	**	**winter**	0.569	**
*Leptocylindrus mediterraneus*	DIAT	**autumn**	0.327	**			
*Diploneis* spp.	DIAT	**autumn**	0.337	**	**winter**	0.349	**
*Calciosolenia murrayi*	COCCO	**autumn**	0.323	*			
*Chaetoceros decipiens*	DIAT	**autumn**	0.368	*	**winter**	0.369	*
*Thalassiosira* spp.	DIAT	**autumn**	0.284	*			
*Thalassionema frauenfeldii*	DIAT	**autumn**	0.365	*			
*Navicula* spp.	DIAT	**autumn**	0.434	*	**winter**	0.527	**
*Cylindrotheca closterium*	DIAT				**winter**	0.495	**
*Pleurosigma* spp.	DIAT				**winter**	0.503	**
*Calciosolenia brasiliensis*	COCCO				**winter**	0.501	**
*Dactyliosolen phuketensis*	DIAT	**winter**	0.408	**	**winter**	0.243	*
*Bacteriastrum* spp.	DIAT	**winter**	0.477	**			
*Asterionellopsis glacialis*	DIAT	**winter**	0.523	**	**winter**	0.478	**
*Chaetoceros curvisetus*	DIAT	**winter**	0.576	**	**winter**	0.535	**
*Syracosphaera pulchra*	COCCO	**winter**	0.513	**			
*Guinardia flaccida*	DIAT	**winter**	0.420	*			
*Rhizosolenia imbricata*	DIAT	**winter**	0.410	*	**spring**	0.423	**
*Chaetoceros affinis*	DIAT				**winter**	0.501	**

## Data Availability

Dataset available upon request from the authors.

## Supplementary Materials

The following supporting information can be downloaded at: https://www.mdpi.com/article/10.3390/biology13070493/s1, Figure S1. Distribution of sampling effort with gaps periods at coastal and offshore stations; Figure S2. Significant increase in species richness throughout study period confirmed by Mann–Kendall test (tau = 0.408, *p*-value < 2.22 × 10^−16^); Figure S3. Monthly distribution of indices (a) number of species (b) Shannon at coastal and offshore stations; Figure S4. Vertical distribution of indices (a) number of species, (b) Shannon at coastal and offshore stations; Figure S5. Mann–Kendall test of correlation between (A) Dinoflagellate Species richness and insolation (tau = 0.223, *p*-value < 2.22 × 10^−16^), (B) sea surface temperature (tau = 0.175, *p*-value < 2.22 × 10^−16^); Figure S6. Seasonal differences of total chlorophyll *a* during study period with presented p–values among seasons (Wilcoxon test).
