# Supplementary material for: Phytoplankton Assemblage over a 14-Year Period in the Adriatic Sea: Patterns and Trends
Source: Biology (Basel). 2024 Jul 2;13(7):493. doi: 10.3390/biology13070493 (PMC11273836; doi:10.3390/biology13070493)
Supplement: Supplementary file 1 [file biology-13-00493-s001.zip › biology-3061790-supplementary.pdf]

Supplementary material

Phytoplankton Assemblage over a 14-Year Period in the Adriatic Sea: Patterns and Trends

Sanda Skejić [1], Blanka Milić Roje [1,*], Frano Matić [2], Jasna Arapov [1], Janja Francé [3], Mia Bužančić [1], Ana Bakrač [1], Maja Straka [1] and Živana Ninčević Gladan [1]

[1] Institute of Oceanography and Fisheries, Šetalište Ivana Meštrovića 63, 21000 Split, Croatia
[2] Department of Marine Studies, University of Split, Ruđera Boškovića 37, 21000 Split, Croatia
[3] National Institute of Biology, Marine Biology Station Piran, 6330 Piran, Slovenia; janja.france@nib.si
* Correspondence: broje@izor.hr

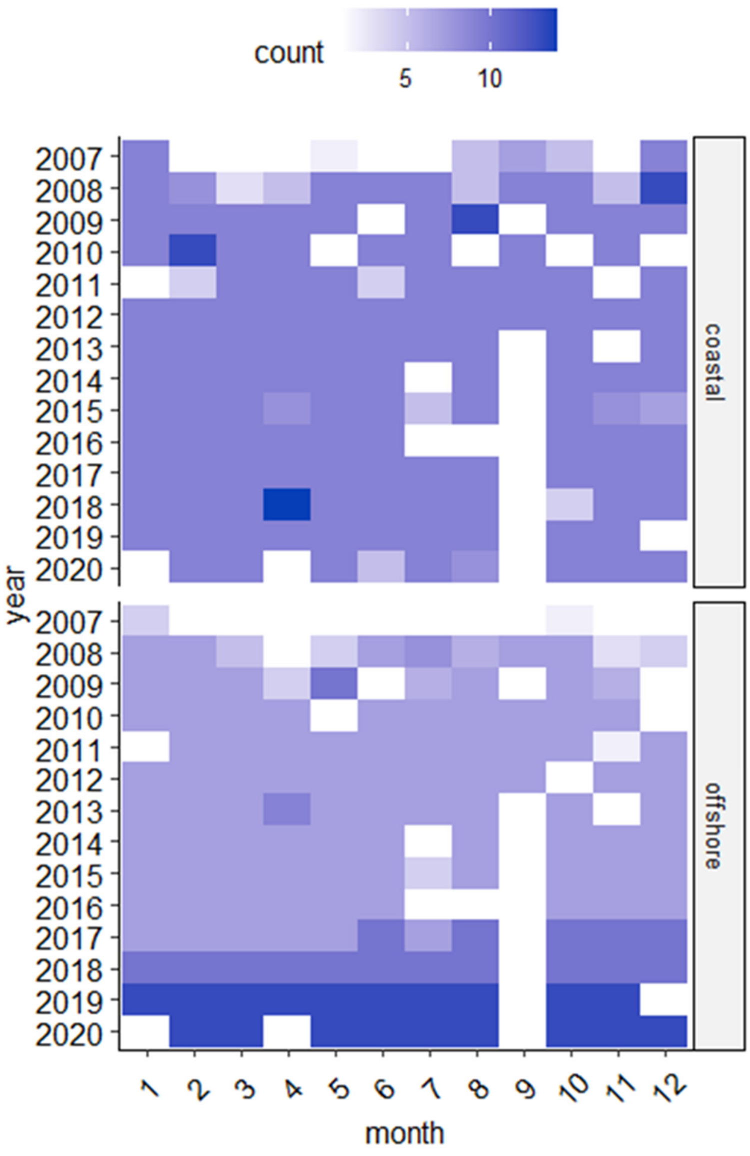

Figure S1. Distribution of sampling effort with gaps periods at coastal and offshore stations.

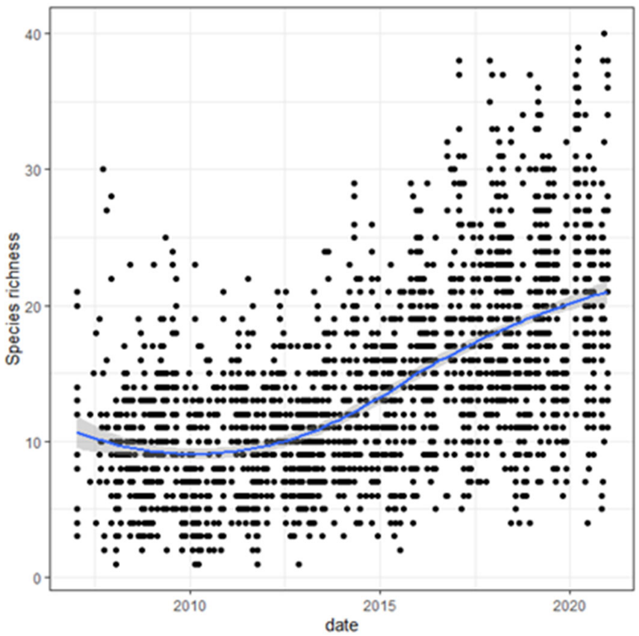

Figure S2. Significant increase in species richness throughout study period confirmed by Mann-Kendall test (tau = 0.408, p-value < $2.22*10^{-16}$).

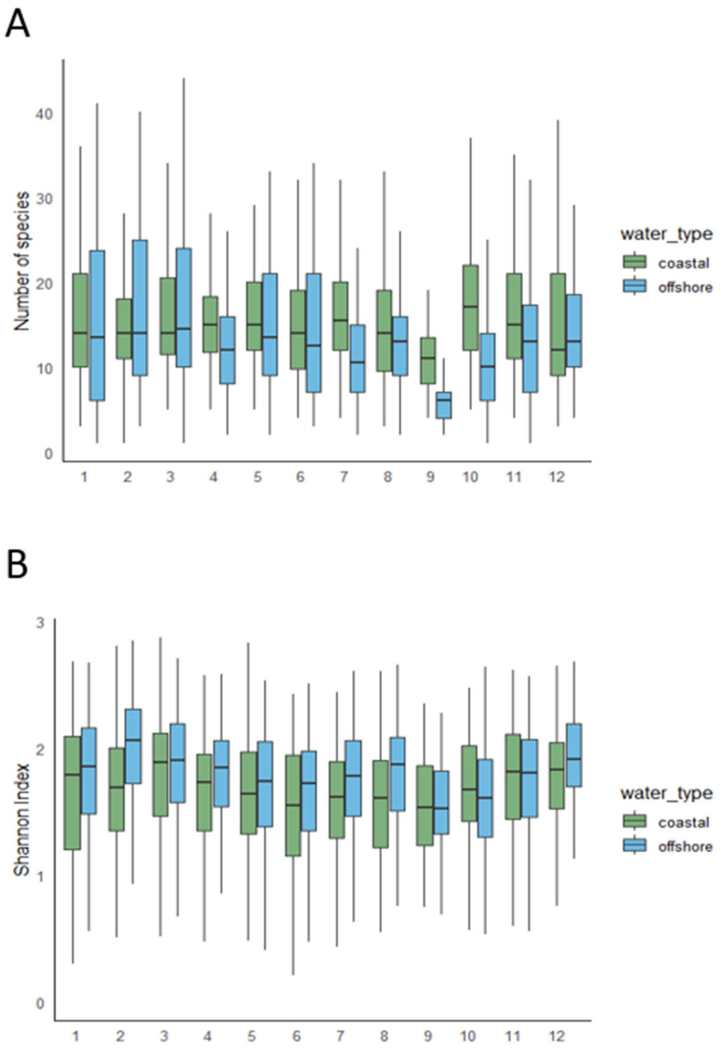

Figure S3. Monthly distribution of indices a) number of species b) Shannon at coastal and offshore stations.

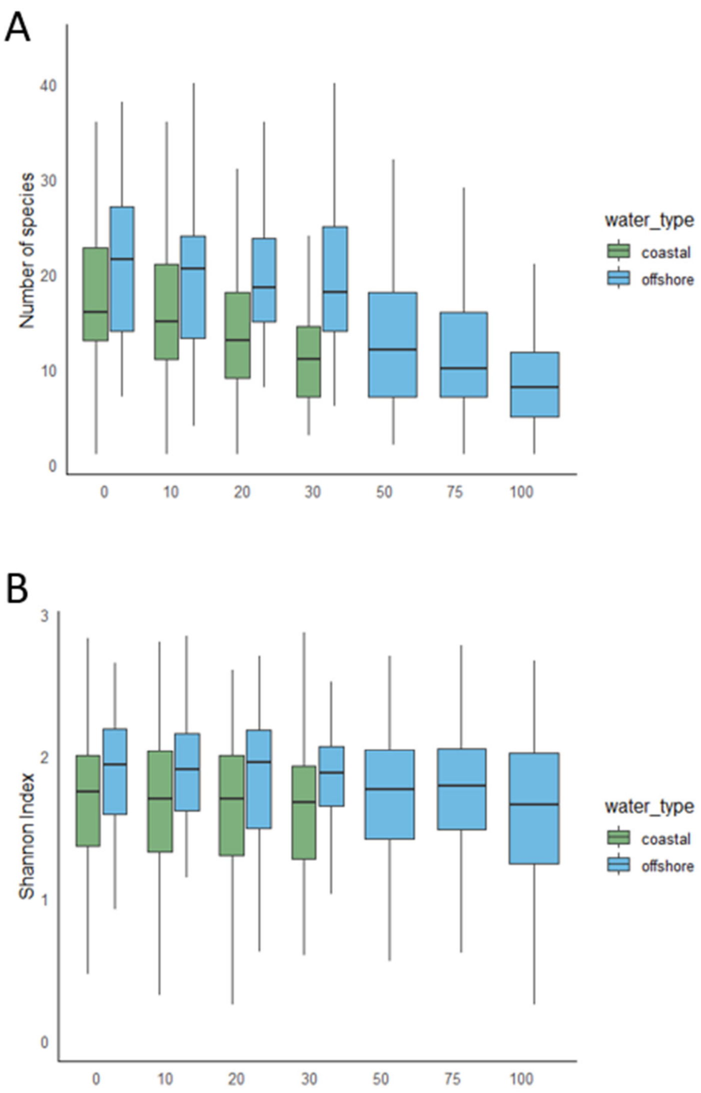

Figure S4. Vertical distribution of indices a) number of species, b) Shannon at coastal and offshore stations.

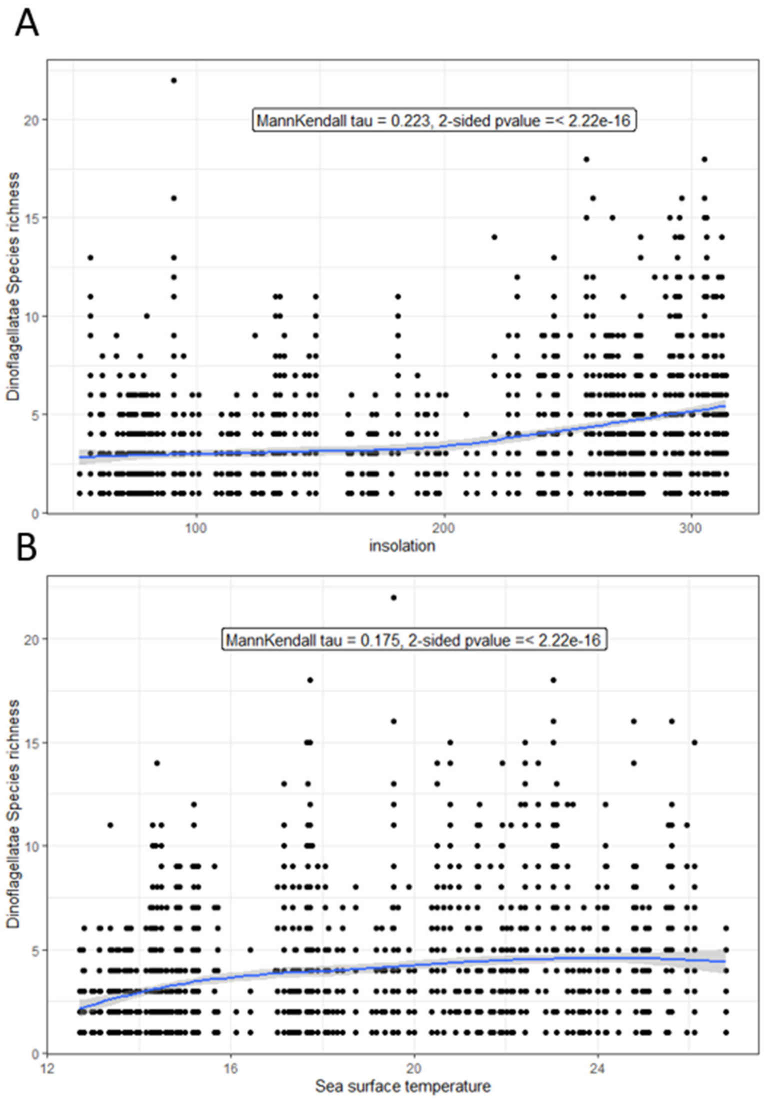

Figure S5. Mann- Kendall test of correlation between (A) Dinoflagellate Species richness and insolation (tau = 0.223, p-value <2.22*10^{-16}), B) sea surface temperature (tau = 0.175, p-value <2.22*10^{-16}).

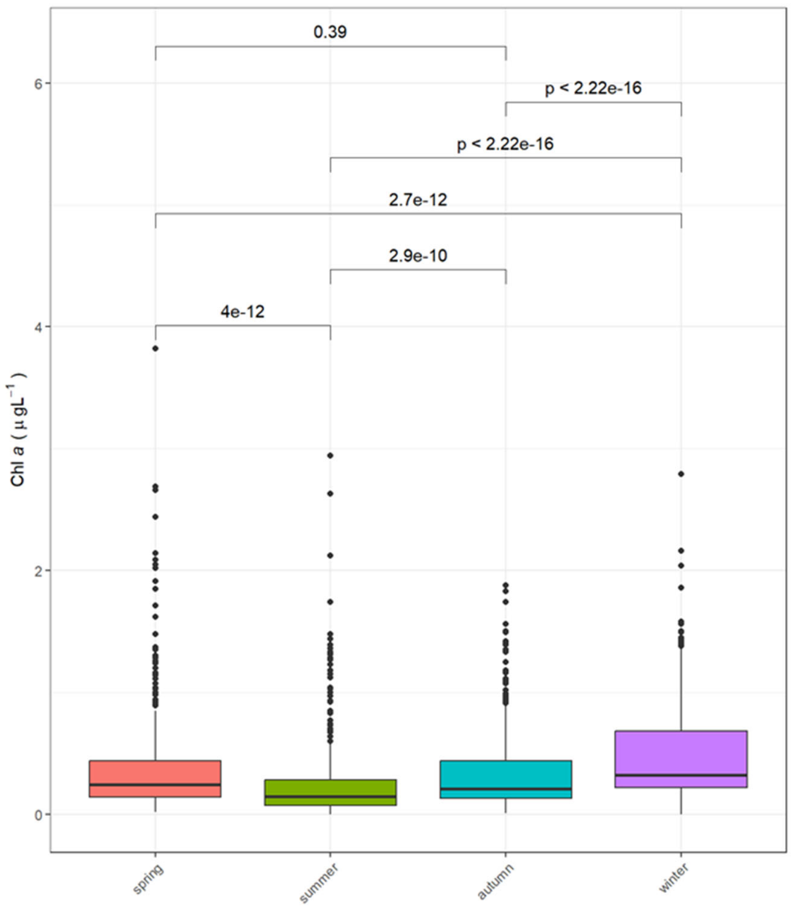

Figure S6. Seasonal differences of total chlorophyll a during study period with presented p-values among seasons (Wilcoxon test).
